# Effect of Age and Sex on Gait Characteristics in the Korean Elderly People

**Published:** 2018-05

**Authors:** Yang Sun PARK, Ji-Won KIM, Yuri KWON, Moon-Seok KWON

**Affiliations:** 1. Dept. of Physical Education, College of Performing Arts and Sports, Hanyang University, Seoul, Korea; 2. Division of Biomedical Engineering, College of Biomedical and Health Sciences, Konkuk University, Chungju-si, Korea; 3. Division of Sports Sciences, College of Science & Technology, Konkuk University, Chungju-si, Korea

**Keywords:** Gait, Age-related difference, Sex difference, Elderly fall

## Abstract

**Background:**

Incidence of falling in the older-elderly is higher than that of the younger-elderly. In addition, falls occur more in elderly women than in elderly men. However, it is unclear whether age and sex-specific differences exist in gait characteristics of the elderly. Therefore, the aim of this study was to investigate age- and sex-related differences in gait characteristics of the Korean elderly people.

**Methods:**

A total of 75 younger-elderly subjects (age of 65–74 yr; 21 men and 54 women) and 59 older-elderly subjects (age of 75–90 yr; 15 men and 44 women) participated in this study in 2014. All participants walked a distance of 8 m across a GaitRite walkway with self-selected speed. The effects of age and sex on spatiotemporal gait variables in the Korean elderly people were analyzed before and after adjusting height as covariate.

**Results:**

The older-elderly group slowly walked with shorter stride length (*P*<0.05) and step length (*P*<0.05) compared to the younger-elderly, regardless of their height. There was no significant sex difference after adjusting height as covariate, although elderly women walked with shorter stride length (*P*<0.01) and step length (*P*<0.01) than elderly men. The elderly women group walked with more variable stride time (*P*<0.05) and with longer double support (*P*<0.01).

**Conclusion:**

Age-related changes and sex difference among the elderly existed in specific gait variables. Characterizing gait patterns of the Korean elderly people considering both age and sex would be beneficial to assess gait of the elderly with risk of falls for fall interventions.

## Introduction

One-third of the elderly experience falls at least once per year (1–3). The rates of falls rise steadily with age, with the rate of the older-elderly with age of more than 75 yr twice of that of the general elderly population (4). In addition, hip fractures due to falls occur more frequently in the older-elderly (aged >75 yr) than those in the younger-elderly (65–75 yr) (4). Furthermore, falls occur more in elderly women than in elderly men, with the fall rate of women 10% to 49% higher than that of men (5–7).

Up to 70% of falls in the elderly happen during walking (8). Spatiotemporal variables of gait can be analyzed to assess the risk factor of fall. For example, gait speed is one important risk factor for fall. Specifically, each 0.10 m/s decrease in gait speed is associated with a 7% increased falls (9). The stride length of fallers is significantly shorter than that of non-fallers (10).

It is important to know whether higher fall rate of the older-elderly and elderly women is correlated with poorer gait characteristics to understand the falling mechanism and to prevent falls. Therefore, investigation on age-related difference and sex difference within the elderly may bring more important insights on gait assessment, functional training, and fall risk interventions.

Due to aging, declined nervous system and musculoskeletal system may affect gait control. In this regard, many studies have investigated gait pattern of the elderly compared to that of the young. The elderly have decreased stride length, gait velocity, and single leg support time compared to the young (11, 12). The elderly have gait strategy to maintain the dynamic balance by increasing double support time while decreasing gait speed and taking shorter steps (13). However, investigation on age-deteriorated changes in gait characteristics among the elderly is not fully explored yet.

In addition, sex difference may exist in gait patterns since musculoskeletal dimensions (i.e. height) of women are different with those of men (14). Women have shorter stride length and slower gait speed in comparison with men mostly due to their shorter height (15). However, the subjects of that study were confined to the young.

It is currently unclear whether sex differs in gait characteristics among the elderly subjects. Gait patterns may differ between sex regardless of the height in the elderly.

We aimed to investigate the possible age-related changes as well as sex difference in gait characteristics of the Korean elderly people. The aims of this study were to: 1) Determine whether there was any difference in gait variables between younger-elderly and older-elderly, and 2) to investigate whether sex differences existed in specific gait variables.

## Materials and Method

### Subjects

This cross-sectional study was to investigate age and sex-related differences in gait characteristics of the Korean elderly people in 2014. With written informed consent, 134 elderly subjects participated were allocated into a younger-elderly group (n = 75, age of 65–74 yr; 21 men and 54 women) and an older-elderly group (n=59, age of 75–90; 15 men and 44 women). Elderly volunteers were recruited from local elderly communities located in Seoul city, Korea (Table 1).

**Table 1:** Subject characteristics

***Characteristics***	***Younger-Elderly Men (n=21) Mean (SD)***	***Younger-Elderly Women (n=54) Mean (SD)***	***Older-Elderly Men (n=15) Mean (SD)***	***Older-Elderly Women (n=44) Mean (SD)***
Age (yr)	70 (2.9)	70 (2.8)	80 (3.6)	78 (2.6)
Age range (yr)	65–74	66–74	75–90	75–86
Height (cm)	164.4 (6.3)	153.4 (6.1)	161.6 (7.1)	150.5 (5.9)
Weight (kg)	67.1 (7.1)	58.6 (8.8)	62.8 (7.7)	56.3 (8.3)
BMI (kg/m^2^)^*^	24.8 (1.8)	25.2 (3.3)	24.0 (2.2)	24.8 (3.1)
BFR (%)+	24.2 (5.6)	34.9 (7.4)	26.4 (6.5)	35.0 (6.3)

* BMI, body mass index

+BFR, Body fat ratio

Participants who could not walk independently and those with any disease affecting their physical activity (i.e., musculoskeletal disease, neurological disease, and cardiovascular disorders) were excluded. All participants’ physical characteristics, i.e., height, weight, body mass index (BMI): and Body fat ratio (BFR): were measured using electronic height and weight measuring machines (SH-9600A, Sewoo system, Korea) as well as a body fat measurement device (Inbody 4.0, Bio-space, Korea). To take accurate measurements, intake of food, alcohol, and caffeine within 2 h of measurement were restricted for all participants.

### Experiments and Outcome Measures

**Fig. 1:**
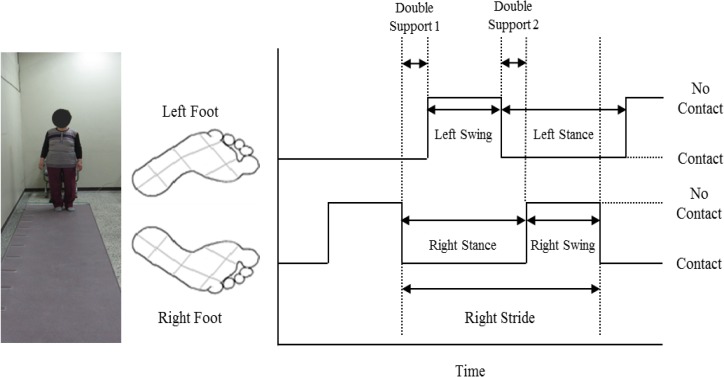
Temporal outcome measures of gait recorded by the GaitRite system

**Fig. 2:**
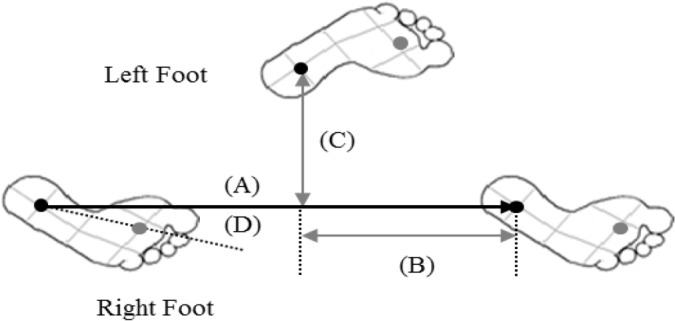
Spatial outcome measures of gait recorded step length (A): stride length (B): base width (C) and toe in/out angle (D) by the GaitRite system. Black dot (heel area) and gray dot (toe area) represent the center of area

Spatiotemporal variables of gait were measured using GaitRite (CIR Systems Inc. Clifton, NJ, USA): a 427 cm long portable walkway mat with an active sensor area at 366 cm long and 61 cm wide. It could detect footfalls as participant walked the length of the mat. The active area contained 13824 pressure sensors arranged in a horizontal grid pattern. Sensors were placed every 1.27 cm. A sampling frequency of 80 Hz and a temporal resolution of 12ms were used. All participants performed warming up with some stretches for 3 min. Then, they practiced walking with self-selected speed on the Gaitrite prior to the experiment. Each participant was instructed to walk straight over 8 m distance at his or her usual comfortable walking speed. In order to exclude the first and last few steps of each trial, subjects started walking from a point 2 m before the start of the mat and stopped at a point 2 m past the mat. Three trials were recorded for each subject. The average of the three was used for subsequent analysis. Many temporal outcome measures such as gait speed, step count, cadence, swing time, stance time, duration of double support, and variability of stride time (Fig. 1) were used (16,17). In addition, step length, stride length, and variability of stride length were used for both rights and left sides as spatial outcome measures (Fig. 2).

### Statistical Analysis

With age-group and sex as independent factors and outcome measures as dependent variables, two-way analysis of variance (ANOVA) was performed to assess the main effect of age-group and sex. In order to exclude the effect of height on some variables, two-way analysis of covariance (ANCOVA) with height as covariate was performed. All statistical analyses were performed using SPSS ver.18 for Windows (SPSS Inc., Chicago, IL, USA). The main effect was considered statistically significant if the *P*-value was less than 0.05.

### Ethical considerations

The present study was approved by the Research Ethics Committee. The study protocol was approved by Bio-Ethics Institutional Review Board of Hanyang University in Seoul, Republic of Korea. Written informed consent was fulfilled by all participants.

## Results

Although the number of women was much more than that of men, there was no significant sex difference in age, suggesting that the subject recruitment was well controlled. Height and weight showed significant difference in age (*P*<0.05) and sex (*P*<0.001). Specifically, height and weight were greater in the younger-elderly (compared to the older-elderly) and in women (compared to men). BFR was significantly greater in women (*P*<0.001). There was no significant difference in BMI.

Descriptive statistics of temporal gait variables in each group are summarized in Table 2.

**Table 2:** The results of ANOVA and ANCOVA for temporal variables

***Gait variables***	***Younger-Elderly Men***	***Younger-Elderly Women***	***Older-Elderly Men***	***Older-Elderly Women***	***Statistical significance of ANOVA***				***Statistical significance of ANCOVA***					
	***Mean (SD)***	***Mean (SD)***	***Mean (SD)***	***Mean (SD)***	***Age difference***		***Gender difference***		***Height effect***		***Age difference***		***Gender difference***	
					***P-value***	***F-value***	***P - value***	***F-value***	***P - value***	***F-value***	***P - value***	***F-value***	***P - value***	***F-value***
Gait speed (cm/s)	109.8 (14.8)	104.7 (15.5)	102.6 (15.0)	100.0 (18.0)	0.038^*^	4.39	0.247	1.351	0.060	3.604	0.151	2.084	0.814	0.055
Cadence (step/min)	108.7 (8.7)	113.7 (9.5)	111.6 (9.1)	113.4 (11.9)	0.519	0.418	0.089	2.940	0.009^**^	6.947	0.843	0.039	0.839	0.041
Swing time Right (s)	0.41 (0.04)	0.38 (0.03)	0.40 (0.03)	0.39 (0.05)	0.679	0.172	0.012^*^	6.57 1	0.013^*^	6.368	0.999	0.000	0.580	0.308
Swing time Left (s)	0.41 (0.05)	0.38 (0.04)	0.40 (0.03)	0.39 (0.05)	0.805	0.061	0.011^*^	6.581	0.004^**^	8.517	0.772	0.084	0.780	0.078
Stance time Right (s)	0.70 (0.07)	0.67 (0.07)	0.69 (0.07)	0.67 (0.07)	0.496	0.466	0.225	1.486	0.070	3.338	0.709	0.140	0.903	0.015
Stance time Left (s)	0.70 (0.07)	0.68 (0.07)	0.68 (0.09)	0.68 (0.08)	0.685	0.165	0.438	0.605	0.017^*^	5.812	0.998	0.000	0.414	0.671
Double support cycle Right (%)	25.4 (4.8)	26.8 (3.7)	25.2 (3.6)	26.7 (3.3)	0.895	0.017	0.055	3.758	0.147	2.132	0.661	0.194	0.553	0.353
Double support cycle Left (%)	24.8 (3.4)	26.6 (3.3)	25.0 (3.4)	26.7 (3.4)	0.748	0.100	0.009^**^	6.891	0.209	1.594	0.970	0.001	0.215	1.554
Variability of stride time Right (s)	0.02 (0.02)	0.03 (0.03)	0.04 (0.03)	0.03 (0.04)	0.180	1.820	0.518	0.420	0.126	2.376	0.308	1.049	0.658	0.196
Variability of stride time Left (s)	0.02 (0.03)	0.05 (0.04)	0.03 (0.04)	0.04 (0.05)	0.680	0.171	0.046^*^	4.060	0.190	1.734	0.506	0.444	0.018^*^	5.725

Notes:

* *P*<0.05,

** *P*<0.01,

*** *P*<0.001, NS: non-significance

Moreover, results of ANOVA and ANCOVA with height as the covariate for temporal variables are shown. In gait speed, the older-elderly walked more slowly compared to the younger-elderly. No significant difference in gait speed was found between elderly men and elderly women. Swing time of elderly women was significantly (*P*<0.05) shorter than that of elderly men. However, the difference was not significant after adjusting height as covariate. In addition, women had significantly (*P*<0.01) longer duration of double support and significantly (*P*<0.05) more variable stride time on the left side. There was no interaction in all variables.

Results of ANOVA and ANCOVA for spatial gait variables are shown in Table 3. In step length and stride length, both significant age effect (*P*<0.01) and sex differences (*P*<0.01) existed.

**Table 3:** Results of ANOVA and ANCOVA for spatial variables

***Gait variables***	***Younger-Elderly Men***	***Younger-Elderly Women***	***Older-Elderly Men***	***Older-Elderly Women***	***Statistical significance of ANOVA***				***Statistical significance of ANCOVA***					
	***Mean (SD)***	***Mean (SD)***	***Mean (SD)***	***Mean (SD)***	***Age difference***		***Gender difference***		***Height effect***		***Age difference***		***Gender difference***	
					***P-value***	***F-value***	***P-value***	***F-value***	***P-value***	***F-value***	***P-value***	***F-value***	***P-value***	***F-value***
Step length Right (cm)	60.5 (6.3)	55.5 (6.7)	55.5 (7.1)	52.6 (7.7)	0.006^**^	7.970	0.005^**^	8.02 2	0.000^***^	19.357	0.045^*^	4.101	0.685	0.165
Step length Left (cm)	60.9 (7.2)	55.0 (6.0)	58.4 (7.9)	53.9 (6.4)	0.002^**^	9.638	0.002^**^	10.049	0.000^***^	30.909	0.029^*^	4.904	0.459	0.551
Stride length Right (cm)	122.3 (13.0)	111.1 (12.2)	111.5 (14.2)	105.8 (14.2)	0.002^**^	9.679	0.002^**^	10.520	0.000^***^	26.58 7	0.026^*^	5.094	0.642	0.217
Stride length Left (cm)	121.8 (13.3)	111.0 (12.1)	111.6 (14.6)	106.0 (14.2)	0.005^**^	8.307	0.002^**^	9.828	0.000^***^	26.032	0.046^*^	4.049	0.594	0.285
Base width Right (cm)	10.6 (3.8)	7.8 (2.5)	10.4 (2.8)	7.9 (2.7)	0.928	0.008	0.000^**^	21.370	0.982	0.001	0.944	0.005	0.000^***^	12.703
Base width Left (cm)	9.9 (3.9)	8.1 (2.7)	10.6 (3.1)	7.8 (2.8)	0.671	0.181	0.000^***^	15.908	0.680	0.171	0.611	0.261	0.005^**^	8.004
Toe in out Right (deg.)	11.4 (5.9)	7.6 (5.8)	15.6 (6.3)	7.4 (7.1)	0.107	2.639	0.000^***^	23.560	0.871	0.026	0.127	2.363	0.000^***^	15.013
Toe in out Left (deg.)	8.4 (5.7)	5.3 (5.1)	13.2 (7.8)	5.8 (7.0)	0.035^*^	4.521	0.000^***^	18.066	0.355	0.862	0.061	3.573	0.000^**^	15.082

Notes:

* *P*<0.05,

** *P*<0.01,

*** *P*<0.001, NS: non-significance

**Fig. 3:**
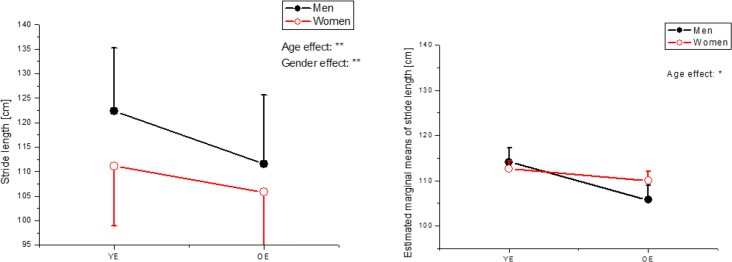
The result of ANOVA and ANCOVA in stride length (YE: younger-elderly, OE: older-elderly)

Specifically, the older-elderly and the elderly women group walked with shorter step length and stride length (Fig. 3). However, after adjusting height, no significant sex difference existed. In contrast, the older-elderly still had significantly (*P*<0.05) shorter step length and stride length than the younger-elderly regardless of their height (Fig. 3).

## Discussion

This study analyzed the gait patterns of the elderly when walking on a walkway. Our results showed age-related difference as well as sex differences in specific gait variables. The main findings of this study were: 1) Age-related differences among the elderly existed in gait speed, stride length, and step length, although height effect was compensated as covariate; 2) Significant sex differences were observed in the duration of double support, variability of stride time.

### Age-related changes

By comparison with the younger-elderly, the older-elderly walked slower with shorter stride length and step length. Interestingly, although height was adjusted as covariate, the older-elderly still had shorter stride length and step length, indicating that stride length and step length of the elderly may be influenced by normal aging regardless of their height. The shorter stride length and step length resulted in reduced gait speed. Indeed, gait speed was significantly correlated to stride length (r=0.81, *P*<0.01) and step length (r=0.82, *P*<0.01). The older-elderly may adapt their gait strategy to maintain dynamic balance by decreasing gait speed and taking shorter stride length and step length for safer and more stable gait.

Age-related difference in gait characteristics was investigated by comparing young subjects to the elderly subjects. In the present study, age-deteriorated changes in specific gait characteristics also existed in the elderly. The elderly walked slower with shorter step length and single leg support time in comparison with the young (11,12). Double support time was increased more in the elderly than in the young (13). According to results of this study, age-related differences within the elderly also existed in stride length and step length regardless of the height (Table 3). On the other hands, single support time (swing time of contralateral leg) and duration of double support didn’t show age-related differences (Table 2). Slower gait speed is associated with increased risk of falls. In addition, fallers have significant reduction in stride length (18,19) and step length (20) compared to non-fallers. Therefore, it is highly plausible that age-related change in gait speed by taking shorter step in the older-elderly as manifested in this study is closely related to higher risk of falls.

### Sex differences

Regarding sex differences, elderly women walked with shorter stride length and step length as well as shorter swing time compared to elderly men, although there was no significant difference in gait speed. Elderly women need to take more steps with shorter strides and swing time in order to walk at a similar speed. Such sex difference might be due to height difference. In fact, no significant sex difference was found after excluding height (Table 2).

On the other hands, sex-specific differences existed in some gait characteristics unaffected by height. Elderly women tend to have more variable stride time and longer double support only at the left side compared to elderly men, indicating that gait control of elderly women may be more unstable and inefficient than elderly men, particularly at the left side. Variability of stride time has been identified as a reliable variable in the rhythmic stepping mechanism depending on the highest-levels of gait control (21). Moreover, higher variability of stride time reflects inefficient gait control and unsafe gait (22). Double support period is a stabilizing factor during a normal gait cycle (23). Elderly women may increase the double support period to stabilize their inefficient gait with variable stride time.

Sex difference in gait characteristics has been investigated in young subjects (15). There was no significant sex difference among young subjects in the duration of double support. However, that study failed to investigate gait variability. In contrast, the present study verified that sex difference did exist in both duration of double support and variability of stride time.

Community-living elderly with a history of falls has increased gait variability (24). Stride-to-stride variability has been suggested to be a significant predictor of future falls (25). The sex difference in stride time investigated in this study is associated with the sex difference in fall ratio because elderly women have more variable stride time than elderly men. Greater variability is associated with fall experience (24). These might explain why elderly women fall more than elderly men.

However, this study has some limitations. 1) Sample size is small in the elderly. Gait assessment should be tested on a large elderly population so that normative data can be provided in the elderly. 2) This study used only spatial-temporal gait variables. As further study, various gait variables such as body acceleration should be analyzed together.

## Conclusion

Age- and sex-related differences in specific gait characteristics among Korean elderly subjects at age of 65∼90 yr. Older-elderly walked slower with shorter stride length and step length regardless of their height. Elderly women walked with longer double support period and more variable stride time. Gait patterns in the elderly depended on age and sex. Therefore, both age and sex should be considered when performing gait assessment for the elderly. Characterizing gait patterns considering both age and sex are likely to be more effective for gait assessment of the Korean elderly people with risk of falls. This will contribute to intervention strategies to prevent or treat gait abnormalities of the elderly.

## Ethical considerations

Ethical issues (Including plagiarism, informed consent, misconduct, data fabrication and/or falsification, double publication and/or submission, redundancy, etc.) have been completely observed by the authors.
